# Modulation of autophagy by RTN-1C: role in autophagosome biogenesis

**DOI:** 10.1038/s41419-019-2099-7

**Published:** 2019-11-18

**Authors:** Manuela D’ Eletto, Anna Risuglia, Serafina Oliverio, Bisan Mehdawy, Roberta Nardacci, Matteo Bordi, Federica Di Sano

**Affiliations:** 1 Department of Biology, University of Rome ‘Tor Vergata’, Via della Ricerca Scientifica, 00133 Rome, Italy; 2 European Centre for Brain Research, IRCSS Santa Lucia Foundation, Via del Fosso di Fiorano 64, 00143 Rome, Italy; 3 National Institute for Infectious Diseases IRCCS ‘L. Spallanzani’, Via Portuense, 00149 Rome, Italy

**Keywords:** Autophagy, Macroautophagy

## Abstract

The endoplasmic reticulum (ER) is a key organelle fundamental for the maintenance of cellular homeostasis and to determine the cell’s fate under stress conditions. Among the known proteins that regulate ER structure and function there is Reticulon-1C (RTN-1C), a member of the reticulon family localized primarily on the ER membrane. We previously demonstrated that RTN-1C expression affects ER function and stress condition. ER is an essential site for the regulation of apoptotic pathways and it has also been recently recognized as an important component of autophagic signaling. Based on these evidences, we have investigated the impact of RTN-1C modulation on autophagy induction. Interestingly we found that reticulon overexpression is able to activate autophagic machinery and its silencing results in a significative inhibition of both basal and induced autophagic response. Using different experimental approaches we demonstrated that RTN-1C colocalizes with ATG16L and LC3II on the autophagosomes. Considering the key role of reticulon proteins in the control of ER membrane shaping and homeostasis, our data suggest the participation of RTN-1C in the autophagic vesicle biogenesis at the level of the ER compartment. Our data indicate a new mechanism by which this structural ER protein modulates cellular stress, that is at the basis of different autophagy-related pathologies.

## Introduction

Endoplasmic reticulum (ER) is an organelle formed by a continuous membrane system comprising the nuclear envelope as well as the peripheral network of tubules and sheets^1,2^. One of the main features of ER is that it is a dynamic structure with both tubules and sheets continuously forming and collapsing. Several findings demonstrated that there are specific proteins regulating ER shaping and morphology^3^, and among these there are reticulons that have been recently indicated as key molecules which are able to stabilize ER tubules curvature^3^. Moreover, they have a role in the nuclear envelope assembly by a mechanism involving ER morphological restructuring^4^.

Reticulons regulate different cellular functions such as membrane trafficking, inhibition of axonal growth, and apoptosis^5^ and they are involved in the pathogenesis of different neurodegenerative diseases^6^. It has recently been suggested that reticulon proteins may play a role also in autophagic process. For example RTN-3 modulates Bcl-2-Beclin 1 interaction in the ER^7^. Moreover, reticulon-related proteins are now emerging as novel regulators of autophagic processes ^8,9^.

Autophagy is a catabolic process essential for cellular homeostasis, delivering cytoplasmic components, such as protein aggregates and organelles, to the lysosome. This degradation system involves the formation of autophagosome structures containing the “cargo material” and the subsequent fusion with lysosomes. There have been identified several proteins which regulate autophagy, although the origin of the autophagosomal membrane is still poorly characterized. It has been proved that ER plays an important role in this process, providing not only the site for omegasome formation but also the membrane for the phagophore elongation^10^. In particular, it has been recently shown that MAMs (mitochondrial ER associated membranes) compartment represents autophagosome formation preferential sites^11^. We previously demonstrated that RTN-1C induces ER stress pathway^12^ and affects mitochondrial morphology and its activity because of its localization in the MAM’s compartment^13^. It is now quite clear that there is a functional crosstalk between ER stress and autophagy which emerged as systems interconnected dynamically^14,15^. These observations together with the reticulon proteins involvement in the regulation of ER-membrane morphology and shaping prompted us to investigate the role of reticulon protein-1C in the control of autophagic machinery; in particular, we analyzed the effect of the reticulon modulation on autophagic flux. Importantly we demonstrated that change in RTN-1C expression levels results in autophagy modulation characterized by a difference in autopagosomes accumulation and cleavage of LC3 protein. Furthermore, we identified that RTN-1C interacts with LC3 protein and colocalizes with the early autophagosome marker ATG16L1; these observations suggest that the reticulon protein may be part of the autophagic machinery and may play a role in the regulation of autophagosome biogenesis.

## Results

### RTN-1C overexpression positively modulates autophagic flux

**Fig. 1:**
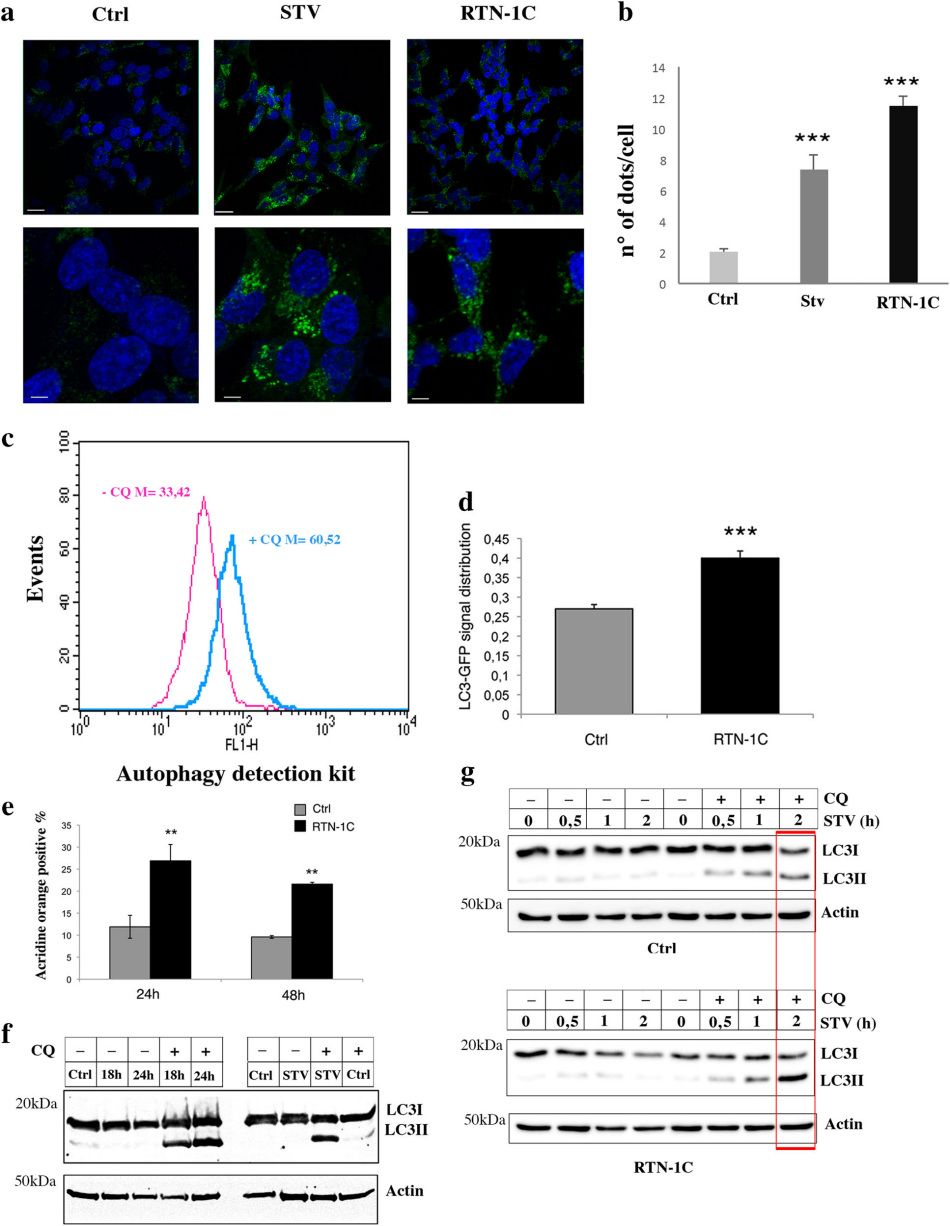
RTN-1C overexpression affects autophagic process. **a** SH-SY5Y control cells (Ctrl), starved for 6 h (Stv) or overexpressing RTN-1C for 24 h (RTN-1C) were stained with anti-LC3 antibody and analyzed by confocal microscopy. Nuclei were controstained by using the fluorescence dye Hoescht-H 33342. Scale bars: 20 μm (upper panels) 5 μm (lower panels). **b** Quantification (means ± SD) of autophagy in SH-SY5Y control cells (Ctrl), overexpressing RTN-1C for 24 h (RTN-1C) or starved for 6 h (Stv), (n = 50). (***) (*P* < 0.001 vs. Ctrl group, Student’s *t* test). **c** Flow cytometry analysis of autophagy in cells overexpressing RTN-1C for 24 h, in the absence or in the presence of 20 μM cloroquine, performed with a Cyto-ID Autophagy Detection Kit. Numbers represent the mean fluorescence intensity. A representative experiments among three is shown. Treatment with lysosomal inhibitor (CQ) increase the fluorescence intensity and is indicative of autophagy activity [17]. **d** SH-SY5Y control (ctrl) or RTN-1C overexpressing cells (RTN-1C) were transiently transfected with LC3-GFP construct for 24 h and analyzed by confocal microscopy. Quantification (means ± SD) of LC3-GFP signal distribution in RTN-1C cells (*n* = 33) compared to control (*n* = 27) is reported. (***) (*P* < 0.001 vs. Ctrl group, Student’s *t* test). **e** SH-SY5Y controls cells or overexpressing RTN-1C for 24 and 48 h were stained with acridine orange and analyzed by flow cytometry. Results are means ± SD of 3 independent determinations. (**) (*P* < 0.01 vs. Ctrl group, Student’s *t* test). **f** Immunoblot analysis of LC3 in SH-SY5Y control cells (Ctrl), starved for 6 h (STV) or overexpressing RTN-1C for the indicated times (18–24 h) in the absence or presence of CQ. Actin was used as loading control. A representative experiment among 3 is shown (**g**) Immunoblot analysis of LC3 in SH-SY5Y control cells (Ctrl) or overexpressing RTN-1C for 24 h (RTN-1C), starved for different times (STV) in the absence or presence of CQ. Actin was used as loading control. A representative experiment among three is shown.

We have previously demonstrated that RTN-1C protein is able to modulate ER stress response through the activation of UPR pathway^12^. Several studies have recently reported a very interesting crosstalk between autophagy and stress signaling arising from intracellular compartments like the ER^16^. Thus, we decided here to test whether RTN-1C modulation may also affect the autophagic machinery. We first looked at LC3 cellular distribution as a marker of autophagosomes formation. Endogenous LC3 staining revealed the typical punctate pattern in WT starved as well as in RTN-1C overexpressing cells indicating LC3 localization in the autophagosomes (Fig. 1a, b). These results suggested a positive correlation between the reticulon expression and autophagy activation. Subsequently we analyzed the impact of RTN-1C overexpression on autophagy by the use of flow cytometry and a Cyto-ID Autophagy Detection Kit^17^; flow cytometry profile clearly demostrated the up-regulation of autophagic activity in the presence of cloroquine after RTN-1C induction as indicated by the increase of the fluorescence signals (Fig. 1c). We found similar results by using an LC3-GFP overexpressing system. Cells with increased levels of autophagic activity had a greater number of autophagosomes and this in turn was associated with a greater variability in pixel intensity; conversely in cells where autophagy was not observed this variation in pixel intensity was lower due to GFP-LC3 signal uniformly distributed throughout the cytosol. Statistical analysis of LC3-GFP signal measurements suggests that RTN-1C overexpression induces significant autophagy, compared to control cells (Fig. 1d). The autophagic process starts with the formation of pre-autophagic vesicles and then progresses to autophagolysosomes through their fusion with lysosomes. Therefore, to verify whether the accumulation of autophagic vesicles observed upon induction of RTN-1C was indicative of an increased autophagic flux, we analyzed the acidification of lysosomes by acridine orange staining of neuronal cells at 24 and 48 h post-RTN-1C induction. As shown in Fig. 1e RTN-1C overexpression markedly elevated the amount of acidic compartments in cells, providing further evidence that the autophagic process was completed. We finally performed western blot analysis of LC3 expression in control cells and in cells where RTN-1C is up-regulated in different nutrient conditions (Fig. 1f, g). Results showed that LC3II band accumulates in RTN-1C overexpressing cells in a time-dependent manner (Fig. 1f); moreover, we observed a marked increase in the autophagic response under starvation condition (Fig. 1g).

**Fig. 2:**
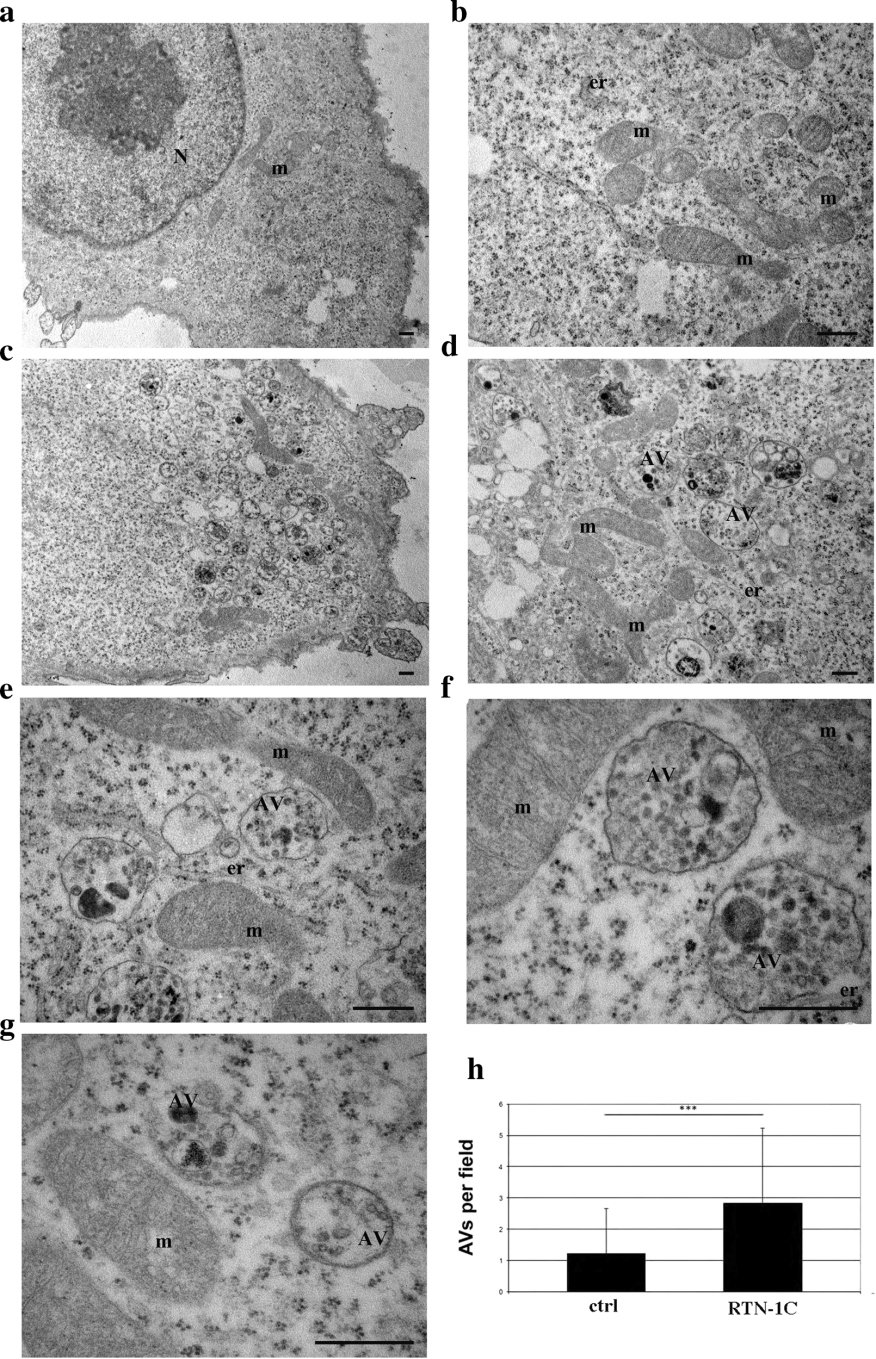
Autophagic vescicles accumulation upon RTN-1C induction. **a**–**g** Ultrastructural analyses of SH-SY5Y neuroblastoma controls cells (**a, b**) or overexpressing RTN1-C for 24 h (**c**–**g**). N nucleus, m mitochondria, AV autophagic vesicles. Scale bars: 1 μm. **h** The number of autophagic vacuoles were counted under the Zeiss EM 900 electron microscope at 12.000x magnification (48 μm^2^) for each treatment conditions. Autophagic vacuoles were classified as autophagosomes when met two or more of the following criteria: double membrane, compartments of 0.5 μm in diameter or larger, luminal uncompacted cytosolic material including organelles, absence of ribosomes attached to the cytosolic side of the membrane. Were examined 70–100 fields per treatment condition and value are expressed as AVs per field. Finally, data were averaged to median values ± standard deviation (±SD) and used for statistical analysis. (***) (*P* < 0.001 vs. Ctrl group, Student’s *t* test).

In order to deeply analyze RTN-1C capability to induce autophagy we performed an electron microscopy analysis; we detected a remarkable autophagosomes accumulation in cells overexpressing RTN-1C (Fig. 2), definitively demonstrating the link between RTN-1C up-regulation and autophagy induction.

**Fig. 3:**
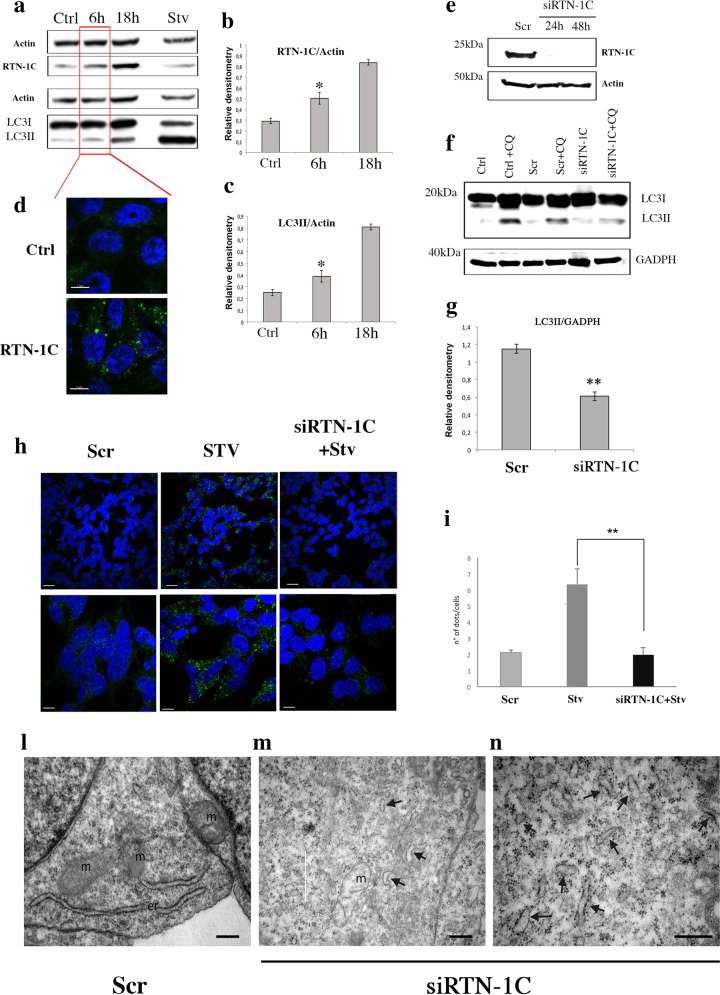
Effect of RTN-1C down regulation on autophagy and endoplasmic reticulum morphology. **a** Immunoblot analysis of RTN-1C and LC3 in SH-SY5Y control cells (Ctrl), overexpressing RTN-1C for the indicated times (6–18 h) or starved for 6 h (Stv) in the presence of 20 μM cloroquine. Actin was used as loading control. A representative experiment among 3 is shown. **b**, **c** Densitometric analysis of RTN-1C (B) and LC3II (C) expression in SH-SY5Y control cells (Ctrl) and overexpressing RTN-1C for the indicated times (6–18 h). (*) (*P* < 0.05 vs. Ctrl group, Student’s *t* test). **d** SH-SY5Y control cells (Ctrl) and overexpressing RTN-1C for 6 h (RTN-1C) were stained with anti-LC3 antibody and analyzed by confocal microscopy. Nuclei were controstained by using the fluorescence dye Hoescht-H 33342. Scale bars: 7 μm. **e** Immunoblot analysis of RTN-1C protein levels in SH-SY5Y wild-type cells treated with scramble siRNAs (Scr) or siRNA specific for RTN-1C at the indicated times. Actin was used as loading control. A representative experiment among three is shown. **f** Immunoblot analysis of LC3 protein in SH-SY5Y control cells (Ctrl), in cells treated with scramble siRNA (Scr) and treated with siRNA for RTN-1C for 24 h (siRTN-1C) in the absence or presence of CQ. GADPH was used as loading control. A representative experiment among three is shown. **g** Densitometric analysis of LC3II expression in control cells (Scr), and in cells treated with siRNA for RTN-1C for 24 h (siRTN-1C) in the presence of CQ. Results represent the mean ± SD of three independent determinations. (**) (*P* < 0.01 vs. Scr group, Student’s *t* test). **h** SH-SY5Y control cells (scramble, Scr) starved for 6 h (Stv) or treated with siRNA for RTN-1C for 24 h and starved for 6 h (siRTN-1C + Stv) were stained with anti-LC3 antibody and analyzed by confocal microscopy. Scale bars: 20 μm (left panels), 7 μm (right panels). Nuclei were controstained by using the fluorescence dye Hoescht-H 33342. **i** Quantification (means ± SD) of autophagy is reported in the bar graph (*n* = 150). (**) (*P* < 0.01 vs. Stv group, Student’s *t* test). **l**–**n** Ultras*t*ructural analyses of SH-SY5Y neuroblastoma controls cells (scramble, Scr) and cells treated with siRNA for RTN-1C for 24 h (siRTN-1C). Arrows indicate endoplasmic reticulum fragmentation. m mitochondria, er endoplasmic reticulum. Scale bars: 1 μm.

Finally, we analyzed LC3 expression and distribution after short time induction of RTN-1C protein to exclude that the influence of RTN-1C on autophagy induction could be caused by altered proteostasis due excessive protein levels. After 6 h induction when RTN-1C is expressed at moderate levels^18,19^ (Fig. 3a–b) and does not induce ER stress condition (data not shown) we observed the accumulation of LC3II band (Fig. 3a–c) as well as autophagosomes formation (Fig. 3d).

### Autophagy inhibition by RTN-1C silencing

Since we demonstrated that the positive modulation of RTN-1C protein is able to induce autophagy, we then verified whether the reticulon protein may play an essential role in the process. To test this hypothesis we knocked down RTN-1C expression by the use of a siRNA approach (Fig. 3e) to verify its effect on autophagy. We first looked at basal autophagy by western blot analysis of LC3 II accumulation (Fig. 3f, g). The results obtained showed a significative decrease of the lower band, corresponding to LC3II in RTN-1C silenced cells.

Subsequently we analyzed the autophagic response to starvation after reticulon down regulation. Data reported in Fig. 3h clearly indicate that the lack of RTN-1C inhibits significantly autophagic flux, as demonstrated by the lower number of LC3-positive autophagosomes in siRTN-1C starved cells in comparison to controls (Fig. 3i).

Finally, in order to deeply characterize the role of the reticulon protein in the autophagic machinery we performed electron microscopy analysis; siRTN-1C cells revealed a dramatic change in ER morphology which appears strongly fragmented (Fig. 3l–n) suggesting its functional deregulation.

Taken together these data suggest not only that RTN-1C is required to maintain basal autophagy but also to support stress-induced autophagy program.

### RTN-1C modulation affects autophagic machinery

**Fig. 4:**
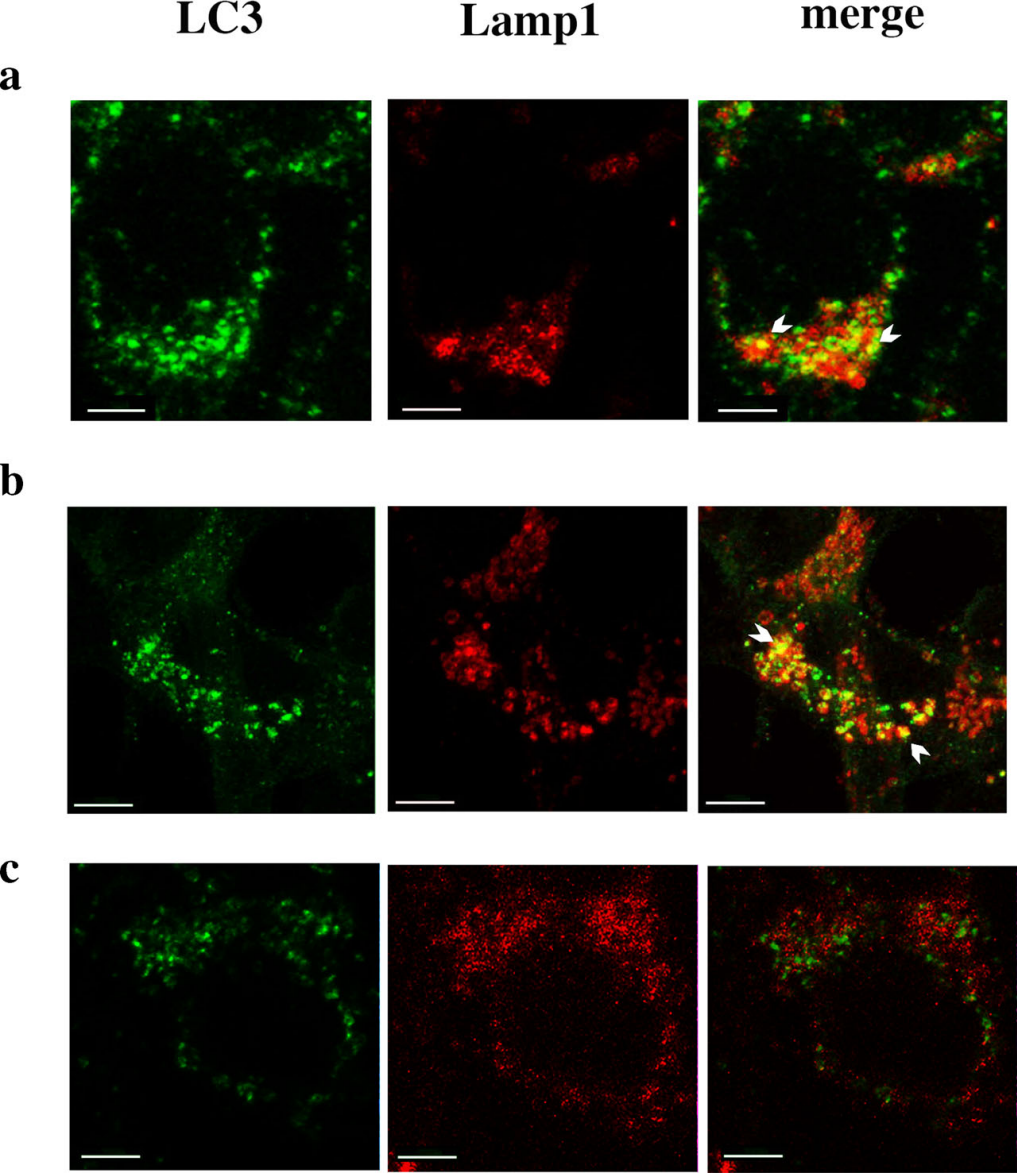
Analysis of autophagosome-lysosome fusion in RTN-1C overexpressing or silenced cells. Double-labeling confocal immunofluorescence microscopy of LC3 (green) and Lamp1 (red) in SH-SY5Y starved cells (**a**), in SH-SY5Y cells overexpressing RTN-1C for 24 h (**b**) and in cells treated with siRNA for RTN-1C for 24 h and starved for 6 h (**c**). The yellow staining in the overlaid images indicates the colocalization (arrowheads). Scale bars: A, C 7 μm, B 5 μm.

In order to better understand the effect of RTN-1C in autophagy induction or inhibition, we then looked to the formation of autophagolysosomes as the final step of the autophagic process. To this aim we used two typical markers for the different cellular compartments; interestingly while in starved (Fig. 4a) and RTN-1C overexpressing cells (Fig. 4b), we observed a proper fusion between autophagosomes and lysosomes during autophagy, by constrast the silencing of reticulon results in the failure of autophagosome–lysosome fusion (Fig. 4c), indicated by the absence of Lamp1 and LC3 colocalization.

**Fig. 5:**
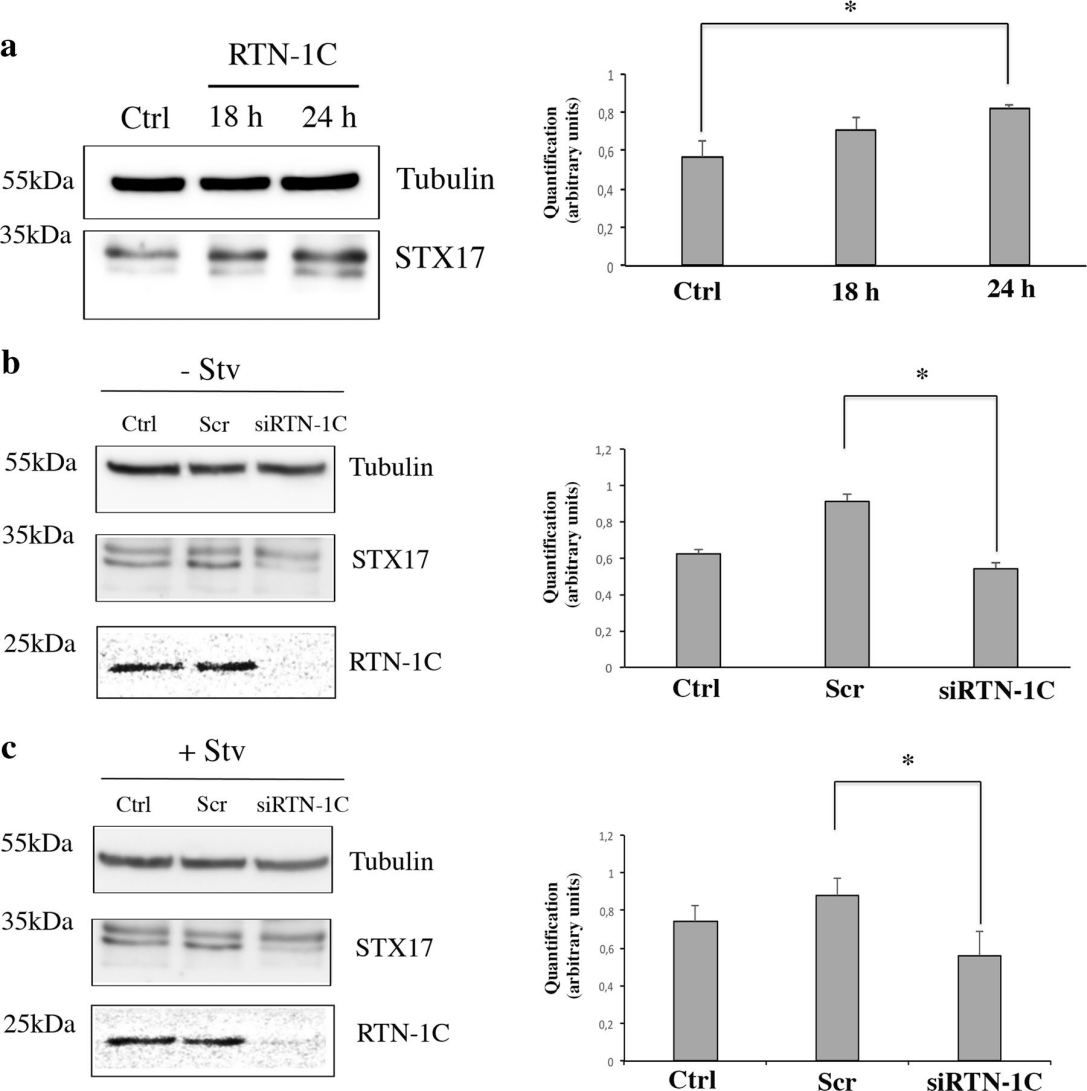
Effect of RTN-1C modulation on syntaxin 17 expression. **a** Immunoblot and densitometric analysis of syntaxin17 in SH-SY5Y control cells (ctrl) or cells overexpressing RTN-1C for the indicated times (18 and 24 h). Tubulin was used as loading control. Results represent the mean ± SD of three independent determinations. (*) (*P* < 0.05 vs. Ctrl group, Student’s *t* test). **b** Immunoblot and densitometric analysis of syntaxin17 in SH-SY5Y control cells (Ctrl), cells treated with scamble siRNA (scr) or treated with siRNA for RTN-1C for 24 h (siRTN-1C). Tubulin was used as loading control. Results represent the mean ± SD of three independent determinations. (*) (*P* < 0.05 vs. Scr group, Student’s *t* test). **c** Immunoblot and densitometric analysis of syntaxin17 after 6 h starvation in SH-SY5Y control cells (Ctrl), cells treated with scamble siRNA (scr) or treated with siRNA for RTN-1C for 24 h (siRTN-1C). Tubulin was used as loading control. Results represent the mean ± SD of three independent determinations. (*) (*P* < 0.05 vs. Scr group, Student’s *t* test).

It has been recently demonstrated that Syntaxin 17 (stx17) facilitates autophagosome formation by the recruitment of ATG proteins to ER-mitochondria contact sites, and it is also required for autophagosome–lysosome fusion^20^. When we measured the level of stx17 we found out that it is up regulated in RTN-1C overexpressing cells (Fig. 5a) and down regulated in RTN-1C silenced cells, both in basal and in starved condition (Fig. 5b, c).

### Role of RTN-1C in autophagosome biogenesis

**Fig. 6:**
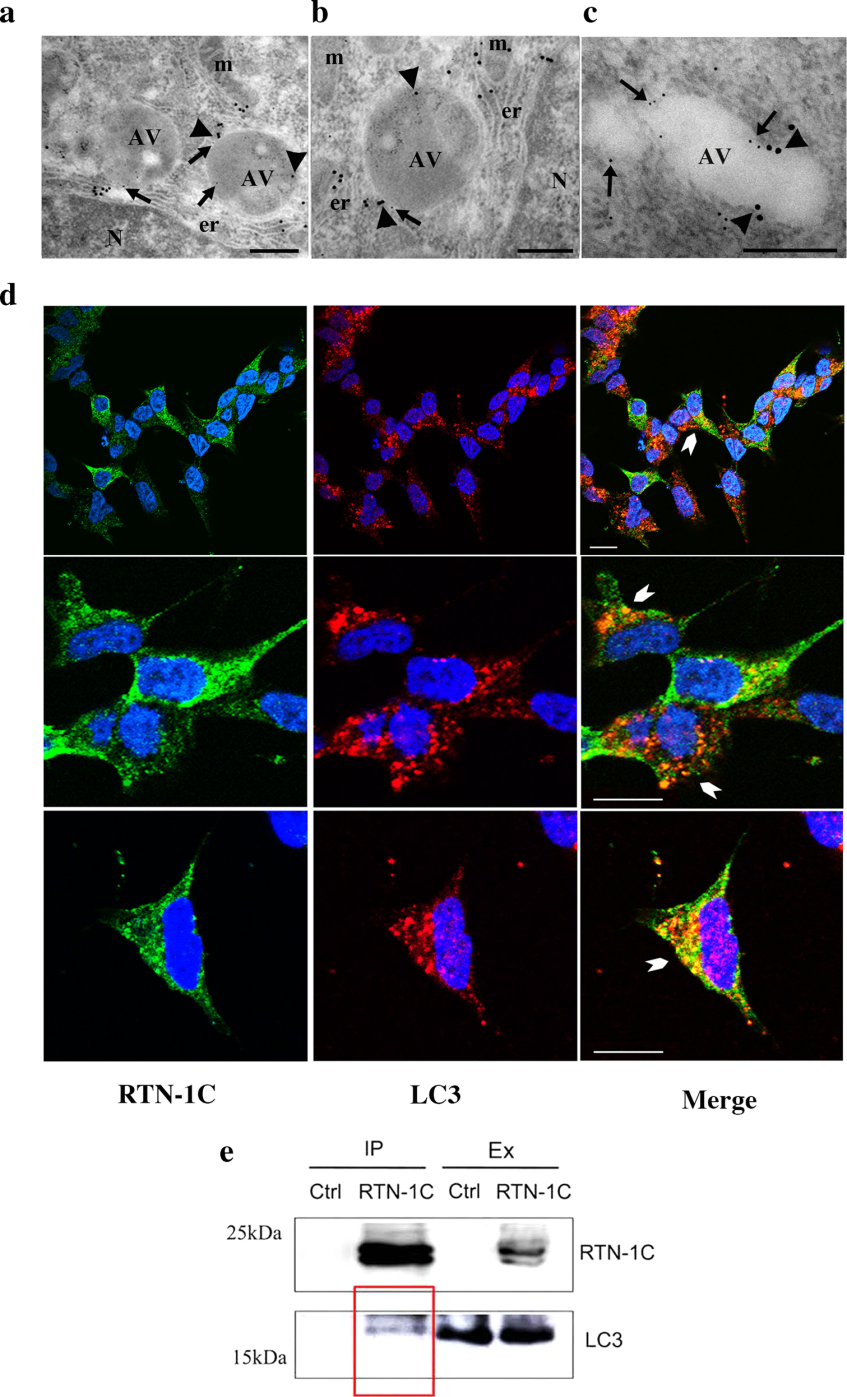
RTN-1C localization on autophagosome membrane. **a**–**c** SH-SY5Y cells overexpressing RTN-1C for 24 h, were subjected to immunogold analysis. A double immunolabeling was performed utilizing anti-RTN-1C and anti-ATG16L1 antibodies, the latter of which was used as early autophagosomes marker. Anti-RTN-1C (5 nm colloidal gold, arrows) and anti-ATG16L1 (15 nm, colloidal gold, arrowheads). AV autophagic vacuoles. Scale bars: 0.5 μm. **d** Double-labeling confocal immunofluorescence microscopy of LC3 (red) and RTN-1C (green) in SH-SY5Y cells (transiently transfected with HA-RTN-1C construct). The yellow staining in the overlaid images indicates the colocalization (arrowheads). Scale bars: 10 μm. **e** Cell lysates from SH-SY5Y wild-type (ctrl) and SH-SY5Y RTN-1C overexpressing cells (transiently transfected with HA-RTN-1C construct) (RTN-1C) were immunoprecipitated with anti-HA antibody and subjected to SDS-PAGE and immunoblotting with an anti-HA antibody (upper panel) and anti-LC3 antibody (lower panel). IP Immunoprecipitate, Ex cell extracts.

On the basis of the results obtained so far and considering the role of RTN proteins in the control of ER structure, we speculated that RTN-1C may take part in autophagy through a possible involvement in the biogenesis of autophagic vesicles from the ER compartment. In order to test this hypothesis, we first analyzed the presence of the reticulon protein on autophagosome membrane by colocalization studies with two autophagosome markers (ATG16L1 and LC3) through electron and fluorescent microscopy respectively. Immunogold analysis showed that ATG16L1 immunolabeling was preferentially localized in structures recognizable as autophagic vacuoles (AV) and importantly RTN-1C immunolabeling was present around the AVs and co-localized with ATG16L1 (Fig. 6a–c).

**Fig. 7:**
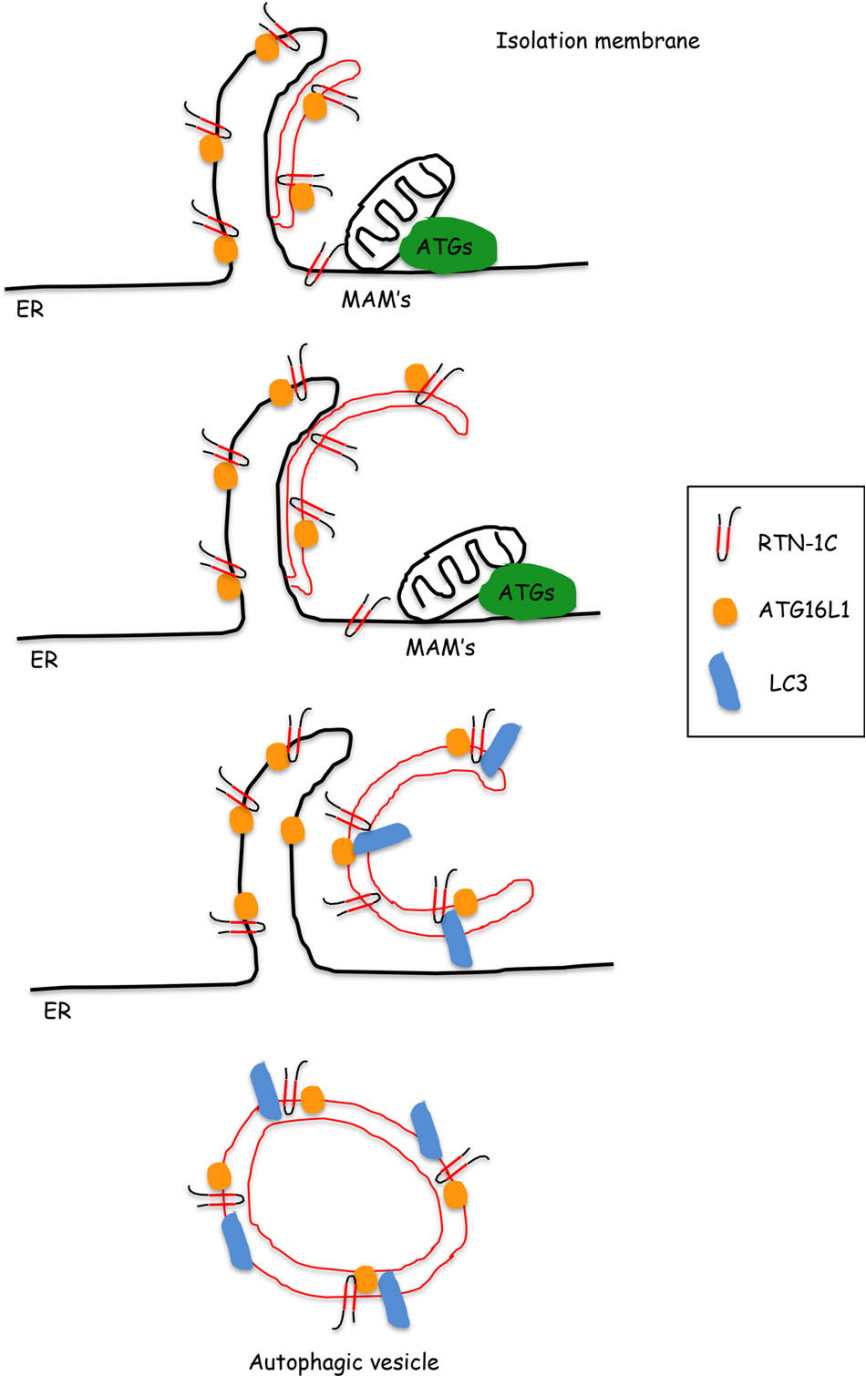
Proposed RTN-1C partecipation in autophagosomes biogenesis. In this model RTN-1C could support the elongation and the curvature of the forming isolation membrane. According to this model RTN-1C, as a protein stabilizing the curvature of the ER tubules, may take part in the autophagic membrane scaffold during autophagosomes formation.

In addition by immunofluorescence studies we found that after autophagy induction RTN1-C co-localized with LC3II on autophagosome membranes (Fig. 6d). Co-immunoprecipitation studies demonstrated the interaction between RTN-1C and LC3 protein confirming the presence of both proteins on the membrane of autophagosomes (Fig. 6e). Computational analysis of RTN-1C aminoacidic sequence highlighted the presence of a putative LIR (*LC3 Interacting Region*) motif (Supplementary Fig. 1a), which is conserved among LC3 interacting proteins. However, RTN-1C interaction with LC3 protein was not affected by the mutation of this domain (Supplementary Fig. 1b). Thus on the basis of these data we propose a model in which RTN-1C, as structural protein localized on the ER membrane and as key component of MAM’s subdomain, participates in the ER-dependent biogenensis of autophagosome vescicles (Fig. 7).

## Discussion

It is well known that distinct signaling pathways at the ER level may contribute to several stress responses linked to protein folding, calcium homeostasis and autophagy^21^. Sustained ER stress and dysfunction of autophagy are associated with many human diseases. Autophagy is a lysosome-mediated catabolic process in which cells eliminate damaged or aged, long-lived proteins and organelles like ER, mitochondria or ribosomes^22^. Thus autophagy plays a crucial role in maintaining cell survival under multiple stresses, restoring cellular homeostasis^23^. Reticulon-1C protein belongs to the reticulon family, a group of ER resident molecules which is particularly expressed in the nervous system, implicated in several cellular function including ER stress, differentiation, cell death and autophagy^5^. These proteins, as structural components of the ER membranes, play a key role in the control of ER shaping and morphology^3^. Autophagic flux is affected by many factors including ER stress^24^. In this context as we have previously demonstrated that modulation of RTN-1C protein is involved in the regulation of ER stress^12^ we investigated here the effects exerted by RTN-1C on autophagic flux. We found evidences that changes in the expression level of the reticulon protein correlates directly with autophagy suggesting that it is involved in the regulation of this process. Interestingly it has been recently demonstrated that FAM134, a reticulon-domain containing protein, is involved in the control of ER-phagy^8^. We observed autophagosomes accumulation upon RTN-1C induction, while, the reticulon protein knockdown resulted in a significant decrease in the number of autophagic vesicles, indicating a direct correlation between the ER-anchored protein levels and the ER-membrane remodeling, required for autophagosome formation. In line with this, we reported here that RTN-1C silencing results in ER fragmentation and deregulation which correlates with the defect in the formation of autophagosomes that we observed in the absence of the reticulon protein. Interestingly RTN-1C silencing affects both basal and starvation-induced autophagy. It is well known that reticulons have the intrinsic membrane bending capacities^3^, hence an imbalance in the concentration of these proteins in the ER compartment likely affects the autophagy machinery. Supporting this notion we also reported that the expression level of RTN-1C protein has a significative impact on LC3- lipidation as well as on lysosome acidification. Intracellular Ca^2+^ is a key regulator of autophagy^25^, and plays a central role in cellular homeostasis and survival during physiologic and pathologic conditions. Although the ER is the main Ca^2+^storage organelle, it has become clear that calcium is handled by other cellular compartments, in particular mitochondria via microdomains of interaction called mitochondria-associated membranes, MAMs^26^. In mammalian cells MAMs represent preferential sites for autophagosome formation^27^; in fact, the specific knockdown of ER-mitochondria tethering proteins negatively affect autophagosome formation^28^. In this context it is worth noting that we have previously demostrated that RTN-1C protein is a key component of the MAMs compartment and it is able to modulate the physical association between ER and mitochondria^13^. This evidence could in part explain the differences here reported in autophagosome formation between RTN-1C overexpressing and RTN-1C silenced cells. The reticulon protein could facilitate the tethering of mitochondria to the ER and conseguently the formation of the autophagosomal membrane. In RTN-1C overexpressing cells we have also observed mitochondrial elongation^13^, a feature commonly found in starved or mTOR silenced cells^29^; this phenotype is well related and further confirmed the activation of the autophagic process by RTN-1C protein.

Autophagosome fusion with degradative compartments depends on different SNARE proteins^30^ and among these, Stx17 is localized to autophagosomes and mediates fusion with lysosomes^30^. Moreover, a model for autophagosome membrane sources provides that syntaxin 17 facilitates ATG proteins targeting to ER-mitocondria contact sites inducing the formation of autophagic vescicles. Interestingly reticulon-1C has been demonstrated to interact with many SNARE proteins regulating vesicle trafficking events^31^. Our data provide evidence that changes in RTN-1C expression also affect Stx17 levels supporting the results obtained concerning the difference in autophagosome formation and the subsequent fusion with lysosomal compartment.

The cargo matherial delivered into the lytic compartment is mediated by molecules interacting with Atg8/LC3 protein via peptide motifs called LC3-interacting regions (LIRs)^32^ and linking the cargo to the autophagosome. We reported here the capability of RTN-1C to influence the formation of autophagosome and the fusion process with lysosome compartment. On the basis of these results we speculate that RTN-1C may take part to the biogenesis of the autophagic vescicles from the ER, subsequently regulating the downstream events such as the fusion process. In order to further validate this hypothesis we investigated the presence of the reticulon protein on the autophagosome membranes. First we observed the colocalization of RTN-1C with different autophagosomes markers, ATG16L1 and LC3 proteins on autophagosomes during autophagy activation. The presence of reticulon-1C on autophagosomes is further confirmed by its interaction with LC3 protein suggesting its likely participation in a multimolecular complex. Altogether these findings indicate an important role for RTN-1C protein in the modulation of autophagic vescicles formation at the ER level. A role for reticulon proteins in the autophagic process has been previously suggested; in particular it has been demonstrated that in mouse neuroblastoma RTN-3 inhibits autophagy via the enhanced interaction between Bcl-2 and Beclin 1, which favors Bcl-2-mediated inhibition of Beclin 1-dependent autophagy^7^. Despite the similarity in their structural features, reticulons display opposite effects in the regulation of this process. More recently Grumati and colleagues reported a role for RTN-3 protein as a specific receptor for the degradation of ER tubules^9^.

Future studies should define the molecular details dictating the alternative behavior of the different RTN family members in the regulation of autophagy. Nevertheless, we assume that RTN-1C involvement in the regulation of autophagy could be related to the role of reticulons as key molecules, regulating and stabilizing the curvature of the ER tubules^3^ which is definitely an essential process during autophagosome formation.

In conclusion, considering the emerging important role played by the disregulation of the autophagic machinery in different human diseases^33^, our data indicate a novel mechanism by which RTN-1C structural ER protein modulates the neuronal stress at the basis of these pathologies.

## Materials and methods

### Cell culture, transfection, and reagents

SH-SY5Y human neuroblastoma cells were purchased from the American Type Culture Collection (ATCC). The derived clones carrying a tetracycline-regulated RTN-1C expression system (RTN-1C) were previously obtained and grown as described^12^. RTN-1C expression was induced by Doxycycline 1 μg/ml.

HA-RTN-1C expression vector was prepared and transfected into SH-SY5Y cells as previously described^34^. For starvation-induced autophagy, cells were incubated in Earle’s Balanced Salt Solution (Lonza,) for 6 h at 37 °C.

Cloroquine was added in colture medium at the concentration of 20 μM for 2 h.

### RNA-interference “knockdown“ of RTN-1C

For RNA-interference ‘knockdown’ of RTN-1C, SH-SY5Y cells were transiently transfected for 48 h with Custom Select siRNA (small interfering RNA) specific for RTN-1C (siRTN-1C) and a scrambled sequence (scrRNA) (Ambion, Life Technologies Ltd,). Each siRNA was trasfected with Xfect^TM^ transfection reagent (Clontech Laboratories,) according to the manufacturer’s instructions.

### Confocal microscopy

To measure the levels of autophagy, cells were transfected with GFP-LC3 plasmids using lipofectamine (Lipofectamine^TM^ 2000, Invitrogen,). Thirty six to forty eight hours after transfection, the 25 mm cover slides were mounted in a microscope chamber and were loaded with KHB. Images requiring higher spatial resolution of cells transfected with GFP-LC3 plasmids were taken on a cooled interline CCD camera (coolsnapEZ–Photometrics) and a confocal CARV LX spinning disk unit using a 63x objective, with an optical depth of 1 µm. Samples were then excited at 488 nm, with emissions collected at 505-530 nm, while being maintained at 37 ^o^C in KHB (in mM: 10 HEPES, 4.2 NaHCO_3_, 1.18 MgSO_4_, 1.18 KH_2_PO_4_, 118 NaCl, 4.69 KCl, 1.8 CaCl_2_, 11.7 glucose, pH 7.4) (Sigma, Italy). The images were analyzed using the LSM Software. Data were analyzed using the Volocity Software. The analysis program enabled us to calculate the standard deviation of pixel intensity within the fluorescent image. The performed analysis was restricted to the portions of image-containing cells. Cells with increased levels of autophagic activity were shown to have a greater number of autophagosomes in their cytosol, and this in turn was associated with a greater variability in pixel intensity (between the bright autophagosomes and the dark cytosol). However, in cells where autophagy was not observed, GFP-LC3 was uniformly distributed throughout the cytosol, therefore, this variation in pixel intensity was not observed (i.e., SD was low). Moreover, in order to correct differences in overall brightness between different images, since the expression of GFP-LC3 was not the same in all cells, the standard deviation was divided by the mean pixel intensity within the area of the analyzed image.

### Autophagy assay (Cyto-ID Autophagy Detection Kit)

Cells were collected and centrifuged at 250 g for 5 min. After washing in PBS, cells were suspended in Assay Buffer and Stain Dye buffer for 30 minutes in the dark, according to the manufacturer’s instructions (Cyto-ID autophagy detection kit, Enzo). Cells were then analyzed by flow cytometry using a FACScan Flow Cytometer (Becton-Dickinson, USA) and Cell Quest software. The cell-ID^TM^ Green autophagy dye serves as a selective marker of autolysosomes and earlier autophagic compartment. The addition of chloroquine leads to an increase of the green fluorescence signals represented by the shift of the fluorescence peak along the abscissa axis.

### Detection and quantification of acidic vesicular organelles with acridine orange staining

Cells were collected and centrifuged at 250 g for 5 min. After washing in PBS, cells were stained with 1 μg/mL acridine orange for 15 minutes and analyzed by flow cytometry using a FACScan Flow Cytometer (Becton-Dickinson, USA) and Cell Quest software.

### Immunoprecipitation

Cells were washed twice with PBS and were lysed in TAP buffer (10 mM Tris pH 8.0, 150 mM NaCl, 10% Glycerol, 0.5% NP-40). For the preclearing, 1 mg of cellular proteins were incubated with protein G for 3 h at 4 °C with continuous rocking. After this, cellular proteins were immunoprecipitated using anti-HA agarose beads (Sigma-Aldrich) for 3 h at 4 °C with continuous rocking. The beads were boiled in sample buffer at 95 °C for 10 min, and samples were resolved by SDS-PAGE and analyzed by Western blotting.

### Immunofluorescence

Cells were grown on poly-l-lysine-coated sterile glass slide. After medium removal, cells were washed twice in PBS, thereafter fixed in cold Methanol for 5 min, then permeabilized for 10 min with 0.3% Triton X-100. Next, cells were rinsed three times with PBS and nonspecific binding sites were blocked with PBS 2% NGS (Normal Goat serum) for 30 min. Appropriate primary antibodies were dissolved in PBS 2% NGS and added for over night incubation. We used mouse anti-RTN-1C (1:100; Abcam, ab8961), rabbit anti-LC3 (1:200; Cell Signaling, 3868 S), mouse anti-LC3 (1:200; Nanotools, 0321-100/LC3-5F10), rabbit anti-ATG16L1 (1:200; Cell Signaling, 8089), rabbit anti-LAMP1 (1:100, MBL, ab 25630), mouse anti-HA (Boehringer, 1583816), rabbit anti-HA (Sigma, H6908) antibodies. After three washes in PBS, cells were incubated for 1 h with the appropriate anti-mouse and anti-rabbit secondary antibodies (Alexa, Molecular Probes, A11034, A11029, A11037) diluted 1:1000 in PBS 2% NGS. Finally, cell nuclei were stained with Hoechst 33342 (Molecular Probes). Slides were observed and photographed in a Leica TCS SP2 or Olympus IX81 (with FLUOVIEW 1000 confocal laser system) confocal microscope.

### Western blot

For Western blot analysis cells were lysed in RIPA buffer and different amounts of total proteins were separated by electrophoresis through SDS-PAGE gels and blotted into nitrocellulose membranes. Mouse anti-RTN-1C (Abcam, ab8961) was diluted 1:1000 rabbit anti-LC3 (Cell Signaling, 3868 S) was diluted 1:1000, rabbit anti-syntaxin 17 (Abcam, ab116113) was diluted 1:200, rabbit anti-HA (Sigma, H6908) was diluted 1:2000. Rabbit anti-actin (Sigma, A2066) diluted 1:2000, mouse anti-GADPH diluted 1:1000 (Santa Cruz, sc-47724) and mouse anti-tubulin 1:2000 (Sigma, T-4026) were used as loading controls. Secondary antibodies (Biorad, 1721011, 1706515) were diluted 1:5000.

### Electron microscopy

Cells were fixed with 2.5% glutaraldehyde (Sigma-Aldrich) in 0.1 M cacodylate buffer, pH 7.4, for 45 min at 4 °C, rinsed in buffer, postfixed in 1% OsO_4_ in 0.1 M cacodylate buffer, pH 7.4, dehydrated, and embedded in Epon resin (Agar Scientific). Grids were thoroughly rinsed in distilled water, stained with aqueous 2% uranyl acetate for 20 min. and photographed in a Zeiss EM 900 electron microscope. The number of autophagic vacuoles were counted under the Zeiss EM 900 electron microscope at ×12.000 magnification (48 μm^2^) for each treatment conditions. Autophagic vacuoles were classified as autophagosomes when met two or more of the following criteria: double membrane, compartments of 0.5 μm in diameter or larger, luminal uncompacted cytosolic material including organelles, absence of ribosomes attached to the cytosolic side of the membrane. We examined 70–100 fields per treatment condition and value are expressed as AVs per field. Finally, data were averaged to median values ± standard deviation (±SD) and used for statistical analysis.

### Immunogold

Cells were fixed in 2% freshly depolymerized paraformaldehyde and 0.2% glutaraldehyde in 0.1 M cacodilate buffer pH 7.4 for 1 h at 4 °C. Samples were rinsed in the same buffer, partially dehydrated and embedded in London Resin White (LR White). Ultrathin sections were processed for immunogold technique. Grids were pre-incubated with 10% normal goat serum in 10 mM PBS containing 1% bovine serum albumine (BSA) and 0.13% NaN_3_ (medium A), for 15 minutes at room temperature. Sections were then incubated with primary antibody, mouse anti-RTN-1C (Abcam, ab8961) diluted 1:100 in medium A for 1 h at room temperature. After rinsing in medium A containing 0.01% Tween 20 (Merck), sections were incubated in goat anti-mouse IgG conjugated to 15 nm colloidal gold (British BioCell Int. EM.GMHL15), diluted 1:30 in medium A, containing fish gelatine, for 1 h at room temperature. The double immunolabeling has been performed incubating the RTN-1C labeled sections with rabbit polyclonal anti-ATG16L1 (Cell Signaling, 8089) followed by goat anti-rabbit IgG conjugated to 5 nm colloidal gold (British BioCell Int. EM.GAR5), diluted 1:30 in medium A, containing fish gelatine, for 1 h at room temperature. Grids were thoroughly rinsed in distilled water, contrasted with aqueous 2% uranyl acetate for 20 min. and photographed in a Zeiss EM 900 electron microscope.

### Statistical analysis

Statistical analyses were performed using GraphPad Prism 6.0. All data are presented as mean ± SD (standard deviation) from at least three separate experiments. The sample sizes were chosen to allow for statistical significance testing assuming a major effect and a small variation. The variance was similar between the compared groups. The *p* values were calculated with two-tailed unpaired Student’s *t* test as indicated in corresponding figure legends. *p* < 0.05 were considered statistically significant.

## Supplementary information

Supplementary figure 1

Supplementary material

Supplementary material

Supplementary material
