# Supplementary figures and images for: Modulation of autophagy by RTN-1C: role in autophagosome biogenesis
Source: Cell Death Dis. 2019 Nov 18;10(12):868. doi: 10.1038/s41419-019-2099-7 (PMC6861279; doi:10.1038/s41419-019-2099-7)

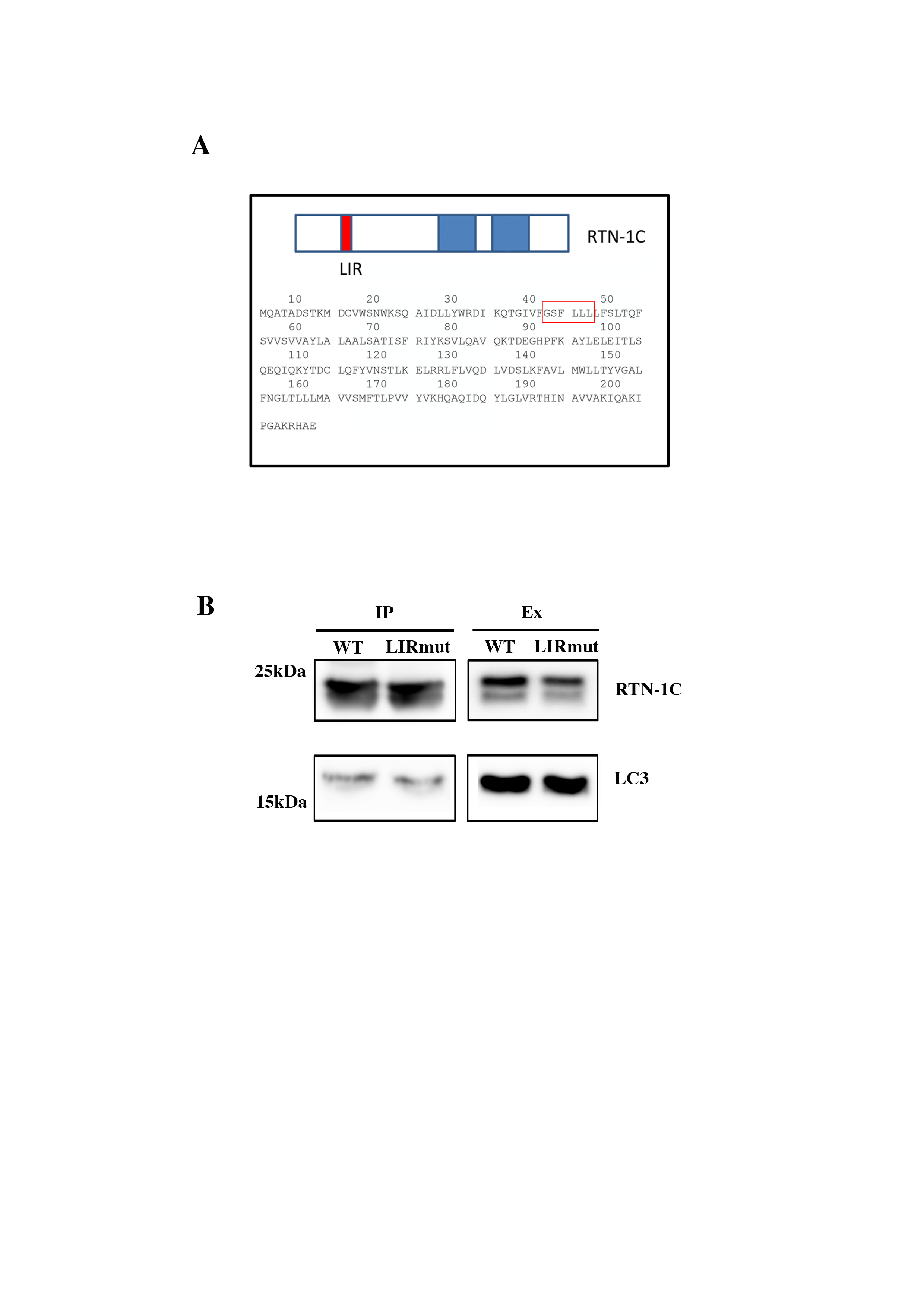

Supplement: Supplementary file 1 — Supplementary figure 1 [file 41419_2019_2099_MOESM1_ESM.tif]
