# Supplementary material for: Modulation of autophagy by RTN-1C: role in autophagosome biogenesis
Source: Cell Death Dis. 2019 Nov 18;10(12):868. doi: 10.1038/s41419-019-2099-7 (PMC6861279; doi:10.1038/s41419-019-2099-7)
Supplement: Supplementary file 2 — Supplementary material [file 41419_2019_2099_MOESM2_ESM.pdf]

Supplementary Materials and Methods

Site specific mutagenesis.

RTN-1C site directed mutagenesis was performed using Quik Change Site-Directed Mutagenesis Kit (Agilent Technologies) according to the manifacture's specifications. Briefly, mutant LIR of the HA-tagged RTN-1C was obtained by PCR using specific primers:

5'-gcagcagcagggcactcccaaacacgatgcccgt-3'

5'-acgggcatcgtgtttgggagtgccctgctgctgc-3'

Immunoprecipitation.

Cells were washed twice with PBS and were lysed in TAP buffer (10 mM Tris pH 8.0, 150 mM NaCl, 10% Glycerol, 0.5% NP-40). For the preclearing, 1 mg of cellular proteins were incubated with protein G for 3h at 4°C with continuous rocking. After this, cellular proteins were immunoprecipitated using anti-HA agarose beads (Sigma-Aldrich) for 3h at 4°C with continuous rocking. The beads were boiled in sample buffer at 95°C for 10 min, and samples were resolved by SDS-PAGE and analyzed by Western blotting.

Supplementary Figure legends

Supplementary Fig. 1: Analysis of LIR domain mutagenesis. (**A**) Schematic diagram of RTN-1C protein showing the identified LIR motif (red box). In blue are indicated the two hydrophobic segments of RTN-1C protein. (**B**) Cell lysates from SH-SY5Y cells transiently transfected with HA-RTN-1C construct wild type (WT) or LIR mutant (LIRmut) were immunoprecipitated with anti-HA antibody and subjected to SDS-PAGE and immunoblotting with an anti-HA antibody (upper panel) and anti-LC3 antibody (lower panel). IP: Immunoprecipitate Ex: cell extracts.
