# Supplementary material for: Modulation of autophagy by RTN-1C: role in autophagosome biogenesis
Source: Cell Death Dis. 2019 Nov 18;10(12):868. doi: 10.1038/s41419-019-2099-7 (PMC6861279; doi:10.1038/s41419-019-2099-7)
Supplement: Supplementary file 3 — Supplementary material [file 41419_2019_2099_MOESM3_ESM.pdf]

DECLARATION OF CONTRIBUTIONS TO ARTICLE

ADMC

Manuscript Number: CDDIS-19-0884RR

Journal Name: *Cell Death & Disease* (the 'Journal')

Proposed Title of the Contribution:

Modulation of autophagy by RTN-1C: role in autophagosome biogenesis. (the 'Contribution)

Author(s):

M. D'Eletto A., Risuglia, S. Oliverio, B. Mehdawy, R. Nardacci. M. Bordi, F. Di Sano

For all *CDD* articles, each person named as an author in the published version must be able to show he or she has contributed substantially to the article.

Authorship credit should be based on 1) substantial contributions to conception and design, acquisition of data, or analysis and interpretation of data; 2) drafting the article or revising it critically for important intellectual content; and 3) final approval of the version to be published. Authors should meet conditions 1, 2 and 3.

Any person who cannot be shown to have made a substantial contribution to the article cannot be listed as an author in the final version. The name of any person who is deemed to have made a minor contribution can, however, appear in the Acknowledgments section of the article.

Please complete the table below to indicate the contributions of all named authors to the manuscript.

Author Full Name:	Specification of Contribution to the Manuscript:
Manuela D'Eletto	she performed western blot analysis, immunoprecipitation and mutagenesis studies
Anna Risuglia	She performed siRNA and immunofluorescence studies.
Serafina Oliverio	She performed flow cytometry and immunofluorescence studies.
Bisan Mehdawy	She performed immunofluorescence experiments
Roberta Nardacci	She performed electron microscopy analysis.
Matteo Bordi	He performed confocal microscopy analysis.
Federica Di Sano	She directed the study and wrote the manuscript.

Please complete the table below to indicate the contributions of all named authors to the figures.

Figure1:

A. Risuglia performed experiments in panels A and B.
S. Oliverio performed experiments in panels C,D and E.
M. D'eletto performed experiments in panels F,G and H.

Figure 2.
R. Nardacci performed experiments in figure 2.

Figure 3. M. D'eletto performed experiments in panels A,B and C.
S. Oliverio performed experiments in panel D.
A. Risuglia performed experiments in panels E, F , G , H and I.
R. Nardacci performed experiments in panels L, M and N.

Figure 4. A. Risuglia performed experiments in figure 4.

Figure 5. A. Risuglia performed experiments in figure 5.

Figure 6. R. Nardacci performed experiments in panels A,B and C.
M. Bordi performed experiments in panels D.
M. D'eletto performed experiments in panels E.

Figure 7. F. Di Sano created figure 7.

Supplementary Figure 1. M. D'eletto performed experiments in panels A and B.

Signed for and on behalf of the Author(s):	Print Name:	Date:
Federica Di Sano Digitally signed by Federica Di Sano DN: cn=Federica Di Sano, o, ou, email=federica.di.sano@uniroma2.it, c=IT Date: 2019.10.04 21:52:49 +01'00'	Di Sano Federica	04/10/2019
